# Tonsillar ectopia in idiopathic scoliosis: does it play a role in the pathogenesis and prognosis or is it only an incidental finding?

**DOI:** 10.1186/1748-7161-4-25

**Published:** 2009-11-12

**Authors:** Kasim Abul-Kasim, Angelica Overgaard, Magnus K Karlsson, Acke Ohlin

**Affiliations:** 1 Faculty of Medicine, Lund University, Lund, Sweden; 2 Division of Neuroradiology, Department of Radiology, Malmö University Hospital, Malmö, Sweden; 3 Department of Orthopaedic Surgery, Malmö University Hospital, Malmö, Sweden

## Abstract

**Background:**

There is an ongoing controversy about the significance of tonsillar ectopia among patients with idiopathic scoliosis (IS).

**Aim:**

To find out if tonsillar ectopia occurs more frequently among patients with IS and if it plays any etiological or prognostic role in IS.

**Study design:**

Retrospective study.

**Methods:**

Retrospective analysis of 155 consecutive spine MRIs (79 patients with IS and 76 controls; aged 7-25 years; 55% were female) with regard to the position of the cerebellar tonsils in relation to foramen magnum and the sagittal diameter of foramen magnum. All images were evaluated independently by two neuroradiologists. Interobserver and intraobserver reliability analysis was performed by calculation of κ-value, intraclass correlation coefficient, and systematic and random errors. The occurrence of tonsillar ectopia among patients with IS and controls was estimated and the association of tonsillar ectopia with different predictors has been tested. Statistical significance was set to P ≤ 0.05.

**Results:**

The interobserver and intraobserver agreement with regard to the occurrence of tonsillar ectopia was almost perfect (κ 0.84 and 0.89, respectively). Tonsillar ectopia was found in 37% of patients with IS compared with 13% among controls (p < 0.001 and odds ratio of 3.8, 95% CI 1.7-8.5). The occurrence of tonsillar ectopia was not associated with the severity of scoliotic deformity (p = 0.85), or rapid progression of scoliosis (p = 0.76). Neurological deficit occurs twice as frequently in patients with tonsillar ectopia as in those with no tonsillar ectopia. Two of five patients with tonsillar ectopia showed improvement of their neurological deficit after the surgical correction of scoliosis.

**Conclusion:**

As tonsillar ectopia is significantly more frequent among patients with IS and may exhibit some prognostic utility in patients with neurological deficit, we forward the hypothesis that tonsillar ectopia *may *play a role in the development of the deformity in some patients with IS. However, occurrence of tonsillar ectopia among 13% of controls precludes stating a definitive role of tonsillar ectopia in the pathogenesis of IS. Some patients with IS, tonsillar ectopia and neurological deficit showed neurological improvement following the surgical correction of scoliosis.

## Background

Idiopathic scoliosis constitutes about 80% of all scoliosis. In the remaining 20% often underlying structural abnormalities can be found. Scoliosis with structural abnormalities are classified according to the nomenclature of the Scoliosis Research Society (SRS), which was developed in 1969, and later modified in 1970 and 1973 [1,2]. Scoliosis was then classified into congenital (neuropathic and osteogenic), neuromuscular, developmental syndromic, and tumor-associated scoliosis [1]. Syringomyelia, Chiari malformations, tethered cord, diastematomyelia and meningocele/myelomeningocele are among the structural abnormalities classified as neuropathic abnormalities.

Chiari malformation was described first by Hans von Chiari in 1891. Four types of Chiari malformations are described in the literature: types I, II, III, and IV. Chiari I malformation is characterized by tonsillar herniation, which is defined as descent of cerebellar tonsils ≥5 mm (≥6 mm in patients younger than 10 years) below the bony border of the foramen magnum [3]. Herniation of cerebellar tonsils of <5 mm (< 6 mm in patients younger than 10 years) below foramen magnum is regarded as tonsillar ectopia.

Etiology and pathogenesis of IS are unknown. However, genetic, musculoskeletal, neurological, hormonal or metabolic factors have been suggested to play possible role in the pathogenesis of IS. Among theories dealing with the pathogeneses of IS are biplanar spinal asymmetry [4], relative anterior spinal overgrowth and biomechanical growth modulation [5,6].

The occurrence of tonsillar ectopia and the allowed level of the cerebellar tonsils in relation to foramen magnum have been a matter of debate. Porter et al [7] reported 50% tonsillar ectopia (tonsils 4 mm below foramen magnum) among patients with idiopathic scoliosis. Cheng et al [8] have expressed lower tolerance in this issue and concluded that any inferior displacement of cerebellar tonsils below the basion and opisthion (BO) line in adolescents should be regarded as abnormal. With this background, we sought to test the hypothesis that tonsillar ectopia occurs more frequently among patients with IS. The second aim of the study was to test the association of tonsillar ectopia with abnormal sagittal diameter of foramen magnum. The third aim was to test the association between the occurrence of tonsillar ectopia in patients with IS and different predictors such as gender, pain, neurological deficit, rapid progression of scoliosis and atypical scoliosis (left thoracic or thoracolumbar curvature).

## Methods

**Figure 1:**
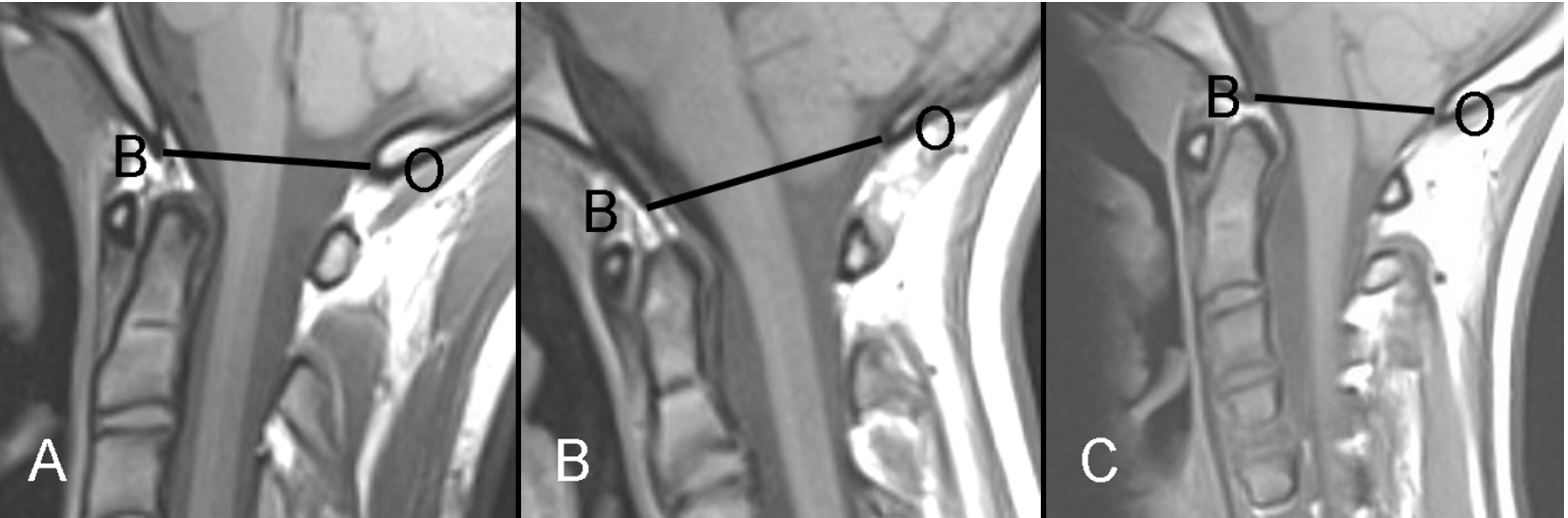
**(A-C): MRI sagittal midline T1-weighted images of three different patients with scoliosis**. The reference lines (BO-lines) connect the basion (B) and opisthion (O). (A) 16 years old boy with IS and no cerebellar ectopia. The cerebellar tonsils are above the BO-line. (B) 16 years old girl with IS showing descent of cerebellar tonsils 4 mm below the BO-line (tonsillar ectopia). (C) 14 years old girl with scoliosis, and Chiari I malformation showing cerebellar tonsils 8 mm below the BO-line (Chiari malformation).

A computerized search about "whole spine magnetic resonance imaging (MRI)" examinations performed in our institution during a 4-year-period (2005-2008) was performed. All patients were examined with 1.5 Tesla MR scanner. The clinical reports of all patients with scoliosis who underwent MRI of the spine were scrutinized. Patients who had scoliosis with a known underlying pathology (i.e. non-idiopathic) were excluded. The search yielded 89 patients referred to the radiology department with the clinical diagnosis of IS. Upon MRI, 10 patients (11.2%) were shown to have neural axis abnormalities (three with syringohydromylia only, one with Chiari I malformation and six with both Chiari I and syringohydromylia). These patients were also excluded from further analysis of this retrospective study. The remaining 79 patients had the final diagnosis of IS and were between the age of 7 and 25 years (only four patients were younger than 10 years) with a mean age of 15.1 ± 3.5 years (mean ± SD). Forty-nine patients were females (62%). Spine MRIs of individuals without scoliosis or neural axis abnormalities of the same age group (n = 76), which were performed within the same period, were considered as a control subjects. The indications for MRI in the control subjects were the following: Non-specific back pain with negative MRI (n = 19), non-specific back pain with positive MRI (n = 9; disc herniation 2, Schmorl's nodules 2, Scheuermann's disease 1, disk degeneration 4), back pain following trauma with negative MRI (n = 19), back pain following serious trauma with skeletal and/or soft tissue injury (n = 8), suspected multiple sclerosis and transverse myelitis (n = 13), tumors (n = 3), spondyloarthritis (n = 4), and pyogenic spondylitis (n = 1). A total of 155 spine MRIs (79 with scoliosis and 76 controls) were included in the analysis of the skull base morphology with regard to the position of the cerebellar tonsils in relation to foramen magnum (basion-opisthion line). The images were evaluated independently by two neuroradiologists who were blinded to the clinical and the radiological reports. T1-weighted sagittal images with 3-mm thickness, were used for the purpose of the evaluations of this study. The position of the cerebellar tonsils was classified as: above foramen magnum, at foramen magnum or below foramen magnum. The cerebellar tonsils located above or at foramen magnum were regarded to have normal position whereas those located below foramen magnum were regarded as ectopic tonsils, Figure 1. The interobserver and intraobserver agreement with regard to the assessment of the position of cerebellar tonsils in relation to foramen magnum was estimated. In cases of interobserver disagreement the status of the cerebellar tonsils was decided in a consensus fashion by joint evaluation of the two neuroradiologists. The anteroposterior diameter of foramen magnum was also measured by the two neuroradiologists. The interobserver agreement in measuring this diameter was also estimated and the final values of the anteroposterior diameter of foramen magnum were the mean value obtained by the two observers. The curve severity was expressed as Cobb angle at the apical vertebra of the major curve. Occurrence of pain, neurological deficit, rapid curve progression, and atypical scoliosis were also recorded (by experienced spine surgeon) for every individual patient with IS. Cobb angle was recorded in every individual patient both at the first and the last radiological control. Rapid progression of the scoliotic deformity was defined as an average increase of the deformity with >1° per month. In patients who underwent surgical correction of scoliotic deformity, the occurrence of neurological deficit postoperatively was specifically sought for.

### Statistical analysis

Statistical analysis was performed with SPSS 17 (originally; Statistical Package for the Social Sciences). Data is presented as proportions (%) or as mean with 95% confidence interval (95% CI) or with standard deviations (SD). The degree of interobserver and intraobserver agreement with regard to the position of cerebellar tonsils was estimated by cross tabulation and calculation of kappa (Ƙ values). The degree of interobserver agreement with regard to the measurements of the sagittal diameter of foramen magnum was estimated by: (1) calculating a two-way mixed model of intraclass correlation coefficient (ICC), and (2) performing a paired sample t-test to calculate the systematic and the random errors for the differences. The interpretations of kappa values and the ICC were done according to the one proposed by Landis [9]. A kappa of 1 indicates total agreement whereas a kappa of zero means poor agreement and indicates that any observed agreement is attributed to chance. Fisher exact test and/or chi-square test were performed to test the association between the occurrence of tonsillar ectopia and categorical variables whereas Mann-Whitney U test was performed studying the association between the occurrence of tonsillar ectopia and continuous variables. Differences with a *P *value ≤ 0.05 were considered statistically significant.

## Results

The two observers agreed about the position of cerebellar tonsils in relation to the bony margin of foramen magnum (BO-line) in 145 (93.5%) out of 155 MRIs included in the analysis. This resulted in an almost perfect interobserver and intraobserver agreement with regard to the occurrence of tonsillar ectopia (κ 0.84, 95% CI 0.74-0.93 and κ 0.89, 95% CI 0.80-0.97 respectively). Eight out of 10 MRIs with interobserver disagreement were considered as normal at the joint evaluation by the two observers.

The interobserver agreement with regard to the measurements of the sagittal diameter of foramen magnum was substantial with ICC of 0.75 (95% CI 0.68-0.81). The systematic and the random error for differences of the measurements performed by the two observers were 0.1 and 0.3 mm, respectively.

The mean value for the age of patients with scoliosis and the controls were 15.1 ± 3.5 and 18.1 ± 4.2 years (mean ± SD) respectively. The range for the age in both groups was 7-25 years. Of 155 patients included in the analysis 85 (55%) were female (49 in the scoliosis group and 36 in the control group), and 70 (45%) were male (30 the scoliosis group and 40 in the control group).

Among 79 patients with IS, 29 (37%) had tonsillar ectopia compared with 10 (13%) among 76 controls, Table 1. This difference was statistically significant, p < 0.001 with an Odds ratio of 3.8 (95% CI 1.7-8.5). However, the occurrence of tonsillar ectopia was not associated with the severity of scoliotic deformity (p = 0.85, Table 2), or rapid progression of scoliosis (p = 0.76, Table 1). Neurological deficit occurred twice as frequently in patients with tonsillar ectopia as in those with no tonsillar ectopia (21% vs 10%, p = 0.31), Table 1. Asymmetrical superficial abdominal reflexes (ASAR) were the most commonly reported neurological deficits in patients with IS (reported in 8 out of 11 patients with neurological deficit). The neurological findings and the associated abnormalities in patients with neurological deficits are shown in Table 3. As shown in Table 1, atypical scoliosis or pain was not a predictor of tonsillar ectopia. There was no statistically significant difference when comparing the sagittal diameter of foramen magnum among the patients with IS and controls, neither between patients with tonsillar ectopia and those with no ectopia, Table 2.

**Table 1:** The association between the occurrence of tonsillar ectopia and different categorical variables.

Tonsillar ectopia			
	Yes	No	P-value
IS	29 (37%)	50 (63%)	
Control	10 (13%)	66 (87%)	< 0.001
Odds Ratio 3.8 (95% CI 1.7-8.5)			
**Gender**			
	Male	Female	
Ectopia	16 (55%)	23 (45%)	
No ectopia	20 (40%)	30 (60%)	0.54

**Neurological deficit**			
	Yes	No	
Ectopia	6 (21%)	23 (79%)	
No ectopia	5 (10%)	45 (90%)	0.31

**Pain**			
	Yes	No	
Ectopia	10 (34%)	19 (66%)	
No ectopia	16 (32%)	34 (68%)	0.82

**Rapid progression of scoliosis**			
	Yes	No	
Ectopia	4 (14%)	25 (86%)	
No ectopia	9 (18%)	41 (82%)	0.76

**Atypical scoliosis**			
	Yes	No	
Ectopia	1 (3%)	28 (97%)	
No ectopia	7 (14%)	43 (86%)	0.24

**Occurrence of tonsillar ectopia among patients with IS only vs those with IS and other associated abnormalities**			

	Yes	No	
Ectopia	19 (37%)	10 (36%)	
No ectopia	32 (63%)	18 (64%)	1.0

**Table 2:** The association between the occurrence of tonsillar ectopia and different continuous variables.

	Mean	Range	P-value
**Age**			
Study population (scoliosis+controls)	16.5 ± 4.1	7-25	
Ectopia	15.6 ± 4.2	7-25	
No ectopia	16.9 ± 4.1	7-25	0.12

**Cobb angle at the major curve**			
Study population (scoliosis+controls)	50.5 ± 16	11-83.9	
Ectopia	50.9 ± 17	15-73.3	
No ectopia	50.3 ± 16	11-83.9	0.85

**Foramen magnum sagittal diameter, mm**			
Study population (scoliosis+controls)	3.5 ± 0.4	2.1-4.5	
IS	3.5 ± 0.4	2.1-4.5	
Control group	3.4 ± 0.4	2.5-4.1	0.22
Ectopia	3.4 ± 0.5	2.1-4.2	
No ectopia	3.5 ± 0.4	2.4-4.5	0.40

**Table 3:** The neurological findings and the associated abnormalities in patients with neurological deficits.

PSN	Neuological findings	Ectopia	Pain	Rapid progression	Atypical scoliosis
26	ASAR	Yes*	No	Yes	No
33	ASAR and hyperactive patellar and Achilles reflexes	Yes	No	No	No
35	ASAR	Yes	No	Yes	No
36	ASAR and hyperactive patellar reflexes	Yes	No	No	No
64	Sensory loss lower limbs and headache	Yes*	No	No	No
79	ASAR	Yes	No	No	No
20	ASAR	No	No	No	No
21	ASAR	No	No	No	No
48	Bilateral clonus: Achilles reflexes	No	No	No	No
67	ASAR	No	No	Yes	No
69	Clonus: Patellar and Achilles reflexes	No	No	No	No

PSN indicates patient study number

ASAR indicates asymmetrical superficial abdominal reflexes

* Patients who showed regression of their neurological deficit following the surgical correction of scoliosis.

Sixty-six percent of patients with tonsillar ectopia and 64% of patients with no ectopia were operated for their scoliotic deformities. Five out of six patients with tonsillar ectopia and neurological deficit were subjected to corrective scoliosis surgery, of which two reported improvement of their neurological deficit after surgery. Only one out of 23 patients with tonsillar ectopia and no neurological deficit preoperatively, reported neurological deficit postoperatively. However, the neurological deficit in that patient had specific dermatome distribution (T8-T10), and was believed to depend on a local extraforaminal nerve injury rather than on the skull base abnormality.

The differences between the occurrence of tonsillar ectopia among patients with IS only and among those with IS and other associated abnormalities (pain, neurological deficit, rapid curve progression, and/or atypical scoliosis) was not statistically significant (36% vs 37%, P = 1.0), Table 1.

## Discussion

This study has shown that tonsillar ectopia was almost three times more common among patients with IS than among controls. However, there was no association between the occurrence of tonsillar ectopia and the severity of the scoliotic deformity, or the curve progression.

The detection of abnormalities that may play role in the pathogeneses of scoliosis is important from the therapeutic and prognostic point of view. The detection of neuropathic abnormalities in patients with scoliosis is considered to be crucial before planning the corrective surgery as some studies have shown that the surgical correction of the spinal deformity in the presence of syringomyelia is associated with high risk for postoperative sequelae such as paraplegia [10,11]. Craniocervical decompression performed before the age of 10 years on patients with Chiari I malformation and scoliosis [12] were shown to improve the spinal curvature or at least stop the curve progression and may thus diminish the need for scoliosis correction surgery. However, spontaneous resolution of syringomyelia and Chiari malformation was reported with non-surgical treatment of scoliosis [13]. As in Chiari malformations [14] neurological deficit occurs more frequently in patients with tonsillar ectopia than in controls. Unlike tonsillar herniation in Chiari I malformation our study showed: (a) no statistically significant difference between the sagittal diameter of foramen magnum in patients with tonsillar ectopia and those with no tonsillar ectopia, Table 2, (b) none of the patients with tonsillar ectopia had the critical sagittal canal diameter at the foramen magnum of 19 mm [15], and (c) there was no report of neurological deficit related to tonsillar ectopia following the surgical correction of scoliosis. In the view of the above facts, we believe that surgical correction of scoliosis in patients with associated tonsillar ectopia can be safely be performed without any preceding intervention of the abnormality of the skull base i.e. tonsillar ectopia. Furthermore, the surgical correction of scoliosis might be beneficial in patients with pre-existing neurological deficit as two out of five such cases reported improvement of their neurological symptoms postoperatively, Table 3. However, Furuya et al showed that posterior fossa decompression improved the neurological deficit in patients with symptomatic tonsillar ectopia [16]. Anyhow, the occurrence of scoliosis among the nine patients with symptomatic tonsillar ectopia included in that study is unknown.

Sun et al [17] showed that tonsillar ectopia was almost six times more common in patients with AIS than in control subjects and that the occurrence of tonsillar ectopia was not correlated with curve severity but with curve pattern (tonsillar ectopia was more common in the thoracic than in lumbar curves). Cheng et al [8] showed association between the occurrence of tonsillar ectopia and curve severity. Furthermore, Cheng et al [18] found that the incidence of tonsillar ectopia in patients with AIS and abnormal somatosensory evoked potentials was 33.3%. This finding together with our observations of frequently occurring neurological deficit among patients with tonsillar ectopia may support a concluding statement that tonsillar ectopia may play a role in the pathogenesis of idiopathic scoliosis.

One limitation of our study is that the patients with IS included in the study population were those from a tertiary referral centre (often patients with scoliosis with Cobb angle > 10° referred for orthopaedic evaluation before eventual therapeutic measures). Hence, the association between the occurrence of cerebellar ectopia and patients with scoliosis with Cobb angle ≤10° remains unknown. As these patients seldom become the subject for MRI-examination or surgery, this issue may possibly remain unanswered. A significant association seems to exist at least between the treatment demanding scoliosis and the occurrence of tonsillar ectopia. Another limitation is that MRI is not performed routinely in all patients with IS in our institution but often reserved primarily to patients with associated symptoms or curve progression that deviates form the expected. However, 28 patients (35% of the whole study population) had no abnormalities other than scoliosis. Among this group of patients the prevalence of tonsillar ectopia was almost the same as that among patients with IS and associated abnormalities (36% vs 37%). Hence, the results of this study can be considered to be representative for patients with idiopathic scoliosis.

## Conclusion

As tonsillar ectopia occurs three times more frequently among patients with IS than in control subjects and as neurological deficit is twice more common in scoliotic patients with tonsillar ectopia than in those without tonsillar ectopia, the authors conclude that tonsillar ectopia *may *contribute to the development of idiopathic scoliosis. However, the occurrence of tonsillar ectopia among 13% of controls restrains us to claim a definitive role of tonsillar ectopia in the pathogenesis of IS. Surgical correction of scoliosis might be beneficial in scoliotic patients with tonsillar ectopia and neurological deficit. Unlike other neuropathic abnormalities associated with non-idiopathic scoliosis such as syringohydromyelia and Chiari I malformation, surgical correction of scoliosis in patients with tonsillar ectopia has not been shown to be complicated by the higher occurrence of postoperative neurological deficit than expected and can thus be safely performed without posterior fossa decompression. Besides playing a possible contributing role in the pathogenesis of IS, tonsillar ectopia may thus exhibit a limited prognostic utility in patients with neurological deficit.

## Competing interests

The authors declare that they have no competing interests.

## Authors' contributions

KAK has contributed to conception and design of the study, acquisition of data, analysis and interpretation of data, drafting the manuscript and has given his final approval of the version to be published.

ANO has contributed to analysis and interpretation of data, revising the manuscript critically for important intellectual content, and has given her final approval of the version to be published.

MKK has contributed to revision of the manuscript critically for important intellectual content, and has given his final approval of the version to be published.

ACO has contributed to conception and design, acquisition of data, revising the manuscript critically for important intellectual content, and has given his final approval of the version to be published.
